# GIS- Based Screening Model of Coastal City Karachi for Plantation of Biofuel Source

**DOI:** 10.1038/s41598-020-61052-9

**Published:** 2020-03-13

**Authors:** Muhammad Jahangir Khan, Syeda Ailya Hasan

**Affiliations:** Department of Earth & Environmental Sciences, Bahria University, Karachi Campus, Karachi, Pakistan

**Keywords:** Environmental impact, Planetary science

## Abstract

Geospatial techniques are mediating in decision making, diversified data management and critical analysis. Jatropha Curcas. is a biodiesel crop and friendly to the regions of saline water environment. This study focuses to map the suitable plantation sites for biodiesel energy crop by using meteorological parameters and satellite imageries of ASTER GDEM and Landsat 8. The thematic layers of soil adjacent to existing vegetation, topographical elevation, slope, land surface temperature, and humidity are created and analyzed with soil types, bareness index and stream orders. Suitability of sites for plantation is a function of these variables which are found to be favorable in the study area. It should be taken into consideration that Jatropha Curcas plantation in Karachi which may contribute in local economic prosperity and support in maintaining heat-sink for the industrialized city.

## Introduction

In recent past, the scientific research intensified on alternate energy resources and sustainable environment. Wide-scale use of fossil fuels in power generation, transportation, industrial units and other sectors are adding excessive pressure on economy and raising geo-environmental problems such as depleting fossil energy resources, engendering imbalance in subsurface masses, greenhouse effect aggravating global warming and ultimately contributing to climate change^1^. As the world struggled to address these issues, biofuels energy flash in the sustainable plans for imminent generations. Global warming can possibly be reduced by adopting bioenergy resources as renewable energy alternatives^2^. According to International Energy Agency (IEA), in the year 2009, the global liquid biofuel production reached up to 83 billion liters which pertains to 1.5 percent of the total fuel used in the transportation sector^3^.

Jatropha Curcas is an unconventional, biodiesel seed plant (second generation biofuel plant) which has been getting attention due the richness of non-edible oil content (about 35 to 48 percent)^4,5^. Jatropha Curcas is a renewable energy plant and friendly to the region of saline water environment^6,7^ like the coastal city Karachi. Due to the steady increase in population, infrastructure, industrial developments and life-style standards, it is apparent that the demand for energy will further increase in the future. The increasing level of greenhouse gases (GHGs) in the atmosphere of mega-cities might be culminated by utilization of ecofriendly fuel. The major cities of Pakistan emit 0.43% of the world’s total GHG emissions^4^, in which significant contribution comes from the populous city Karachi^8^. The alternate energy products like Jatropha Curcas production considerably reduced the greenhouse gas emission (68–89%) and saved energy (65–90%) in comparison to diesel fuel^9^. Jatropha Curcas plant which was originally native to South American countries including Mexico, Mesoamerica^10^ is now being cultivated successfully in some others regions (tropical regions, Nigeria, Pakistan, and India)^11–13^. Contrary, it is also argued that Jatropha Curcas has negative interventions in environment because its plantation can cause deforestation, disturb the equilibrium of local food crops and risk deprivation of farmers from their lands^14^. It is also believed that the water demand of Jatropha Curcas may cause exhaustion of water required for other substantial crops to grow^15^. However, Jatropha Curcas can be planted on waste, degraded, bare or marginal lands that have previously been declared unsuitable for food crop cultivation and thus its use does not threaten the food security^16,17^. Considering that agriculture land is not a special requirement for Jatropha Curcas rather its growth possible on waste land and saline soil etc.^6^. Karachi is the populous coastal city of Pakistan, located on the coast of the Arabian Sea. A pilot study was conducted in Karachi at Pipri Marshaling Yard, Bin Qasim Town, southeast of Karachi, under a joint venture of Pakistan Agriculture Research Council (PARC) and Pakistan State Oil (PSO)^18^. Integration of modern Geographic Information System (GIS) and satellite remote sensing techniques with traditional Knowledge of earth sciences provide substantial solutions in mapping the sites favourable for crops. The recent evolution of satellite technology has been contributed to model extensive earth resources, elucidate best sites, recognizing anomalous pockets. Moreover, the applications of GIS and remote sensing are benefitting in solving several problems related to ground features in low-economic budgets.

The studies^11,12,19^ adopted the GIS based site investigation techniques to recommend the suitable site for plantation of Jatropha Curcas in Thatta (Pakistan), Mysore (India) and Phetchaburi (Thailand). The goal of this study is to assess the Karachi region for plantation of Jatropha Curcas To achieve this ontology, different climatic and terrain parameters were considered along with soil type of the study area by utilizing GIS techniques coupled with satellite images.

An interdisciplinary methodology is designed for locating suitable sites for plantation of Jatropha Curcas in Karachi which helped us to classify the study area in most, moderate and less suitable sites.

## Material and Method

**Figure 1:**
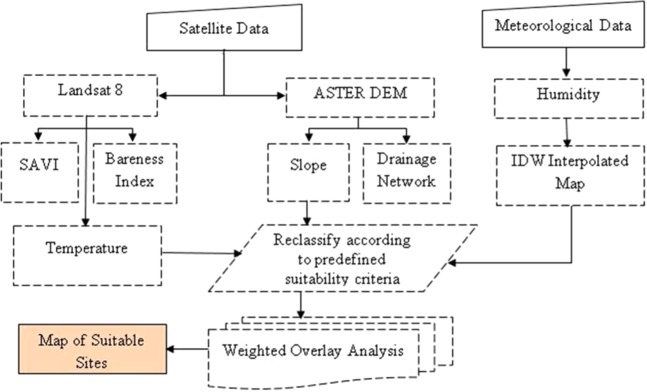
Methodological framework for GIS based suitability model for Jatropha Curcas plantation in Karachi.

We have considered the factors such as land surface temperature, bareness of land, slope, surface elevation derived from remote sensing data and trend of humidity and rainfall to build the suitability criteria. These factors had an impact on the growth of Jatropha Curcas thus recommended to consider for knowing the suitable sites^10,11,16,20,21^. The remote sensing dataset consists GDEM (VNIR spectral bands) of Advanced Spaceborne Thermal Emission and Reflection Radiometer (ASTER) and OLI bands of Landsat 8. The raster data of both satellites gridded at 1 arc-second resolution. The point source data of climatic factors (humidity and precipitation) and soil type maps are gathered from open sources. The integrated workflow is presented in Fig. 1. We have employed ArcMap tools to generate the desired maps and to build suitability model. A mosaic of GDEM tiles was prepared to analyse topography of study area. Moreover, the watershed pathways, slope, and surface elevation were derived from GDEM. The Land Surface Temperature (LST), Soil Adjusted Vegetation Index (SAVI) and Bareness Land Index (BLI) are derived by image processing techniques i.e. spectral band algebra. The point source data of relative humidity is interpolated to prepare a thematic raster layer and to infer the local trends of humidity. LST is a complex mathematical algorithm of land surface emissivity (LSE), thermal brightness of surface, and atmospheric water vapor content^21^. LST is computed at 30 meter resolution from TIRS bands of Landsat 8 by split window algorithm^21^ (Eq. 1)

1 $${\rm{LST}}={{\rm{TB}}}_{10}+{{\rm{C}}}_{1}({{\rm{TB}}}_{10}-{{\rm{TB}}}_{11})+{{\rm{C}}}_{2}{({{\rm{TB}}}_{10}-{{\rm{TB}}}_{11})}^{2}+{{\rm{C}}}_{0}+({{\rm{C}}}_{3}+{{\rm{C}}}_{4}{\rm{W}})(1-{\rm{\varepsilon }})+({{\rm{C}}}_{5}+{{\rm{C}}}_{6}{\rm{W}})\Delta {\rm{\varepsilon }}$$

C0 to C6 = Split-Window Coefficient values given in Table 1.

**Table 1:** SW coefficient values (Skokovic *et al*. 2014^28^ and He *et al*. 2009^29^).

Constant	Value
C_0_	−0.268
C_1_	1.378
C_2_	0.183
C_3_	54.300
C_4_	−2.238
C_5_	−129.200
C_6_	16.400

TB10 and TB11 = brightness temperature of band 10 and band 11

ε = Mean LSE of TIR bands

W = Atmospheric water vapor content

Δ ε = Difference in LSE.

BLI is estimated as an expression of OLI Bands such as red (OLI_4_), near infra-red (OLI_5_) and short-wave infra-red 1 (OLI_6_).

2 $${\rm{BLI}}=({{\rm{OLI}}}_{4}+{{\rm{OLI}}}_{6})-({{\rm{OLI}}}_{5})$$

The difference of NIR and Red spectral bands are sensitive for determination of vegetation cover. SAVI function is derived from OLI bands [red (OLI_4_) and near infra-red (OLI_5_)] by using the Eq. 3

3 $${\rm{SAVI}}=\frac{({\rm{O}}{\rm{L}}{\rm{I}}5-{\rm{O}}{\rm{L}}{\rm{I}}4)}{({\rm{O}}{\rm{L}}{\rm{I}}5+{\rm{O}}{\rm{L}}{\rm{I}}4+{\rm{L}})}\ast (1+{\rm{L}})$$

where L = 0.5 is a constant, characterized for land having intermediate vegetation. A value of zero is considered for high vegetation cover and value 1 is considered for low vegetation cover. Since, there is low vegetative cover in Karachi, thus, we used L = 1 in Eq. 3.

Once the desired thematic layers are derived, a composite set of multi-layers (precipitation, surface temperature, slope, elevation, watershed, humidity) masked by using bare land extent of the study area. The thematic layers are reclassified in order to perform integrated overlay analysis. We tried with various ratios of factors in overlay analysis but gained fair results with equal weightage of the factors. Thus, equal weightage overlay analysis was performed to combine spatially and spectrally distributed layers for generating a final map based on well-defined criteria (Table 2).

**Table 2:** The factors with their suitability criteria for plantation of Jatropha Curcas.

Parameter	Highly Suitable	Less Suitable	Reference
Slope	0–15°	>30°	^27^
Elevation	<1500 m	<0 m and >2150 m	^7^
Land Surface Temperature	20–28 °C	<17 °C and >28 °C	^27^
Relative Humidity	>30%	<38%	^1^
Soil Type	Loamy, sandy or gravelly well drained soil	Clay soil or water logged	^26^

## Results

**Figure 2:**
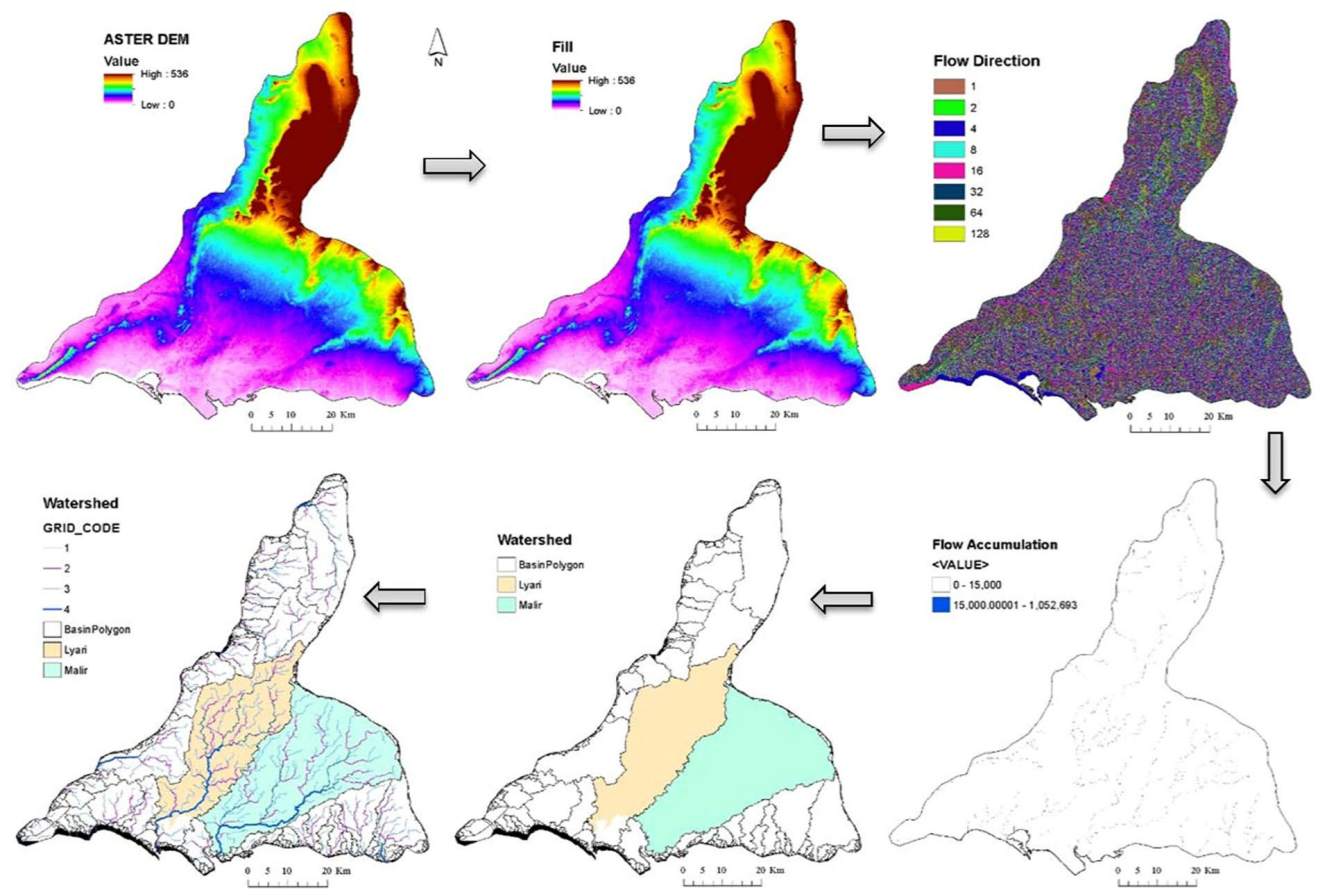
The process of delineation of watershed and drainage network through ASTER GDEM for Karachi. The streams are ordered according to Strahler’s method.

A newly constructed watershed model is presented drainage network in Karachi (Fig. 2), which is an essential ingredient of our suitability model. The drainage network was extracted for Karachi by considering the Strahler’s method applied on ASTER GDEM data using Hydrology toolset of ArcMap. The stream network in the study area is presenting in 1–4 order of streams. The streams linked without any tributaries or the exterior links are assigned an order of 1, referred to as first order streams. As streams of the same order intersect, the stream number increases. Thus, the intersection of two first-order streams creates a second-order stream, just as the intersection of two second-order streams creates a third-order stream, and so on. However, the intersection of two links of different orders will not create a higher order link. Therefore, the intersection of a first-order and second-order stream does not create a third-order stream and instead will retain the order of the highest ordered stream.

The higher order streams delivering water (in this case, the 4^th^ order streams) are considered highly suitable for supply of water to the plantation. The middle order streams (i.e. 2^nd^ and 3^rd^ order streams) are moderately suitable. Whereas, the lowest order streams (1^st^ order streams) are least suitable for Jatropha Curcas.

**Figure 3:**
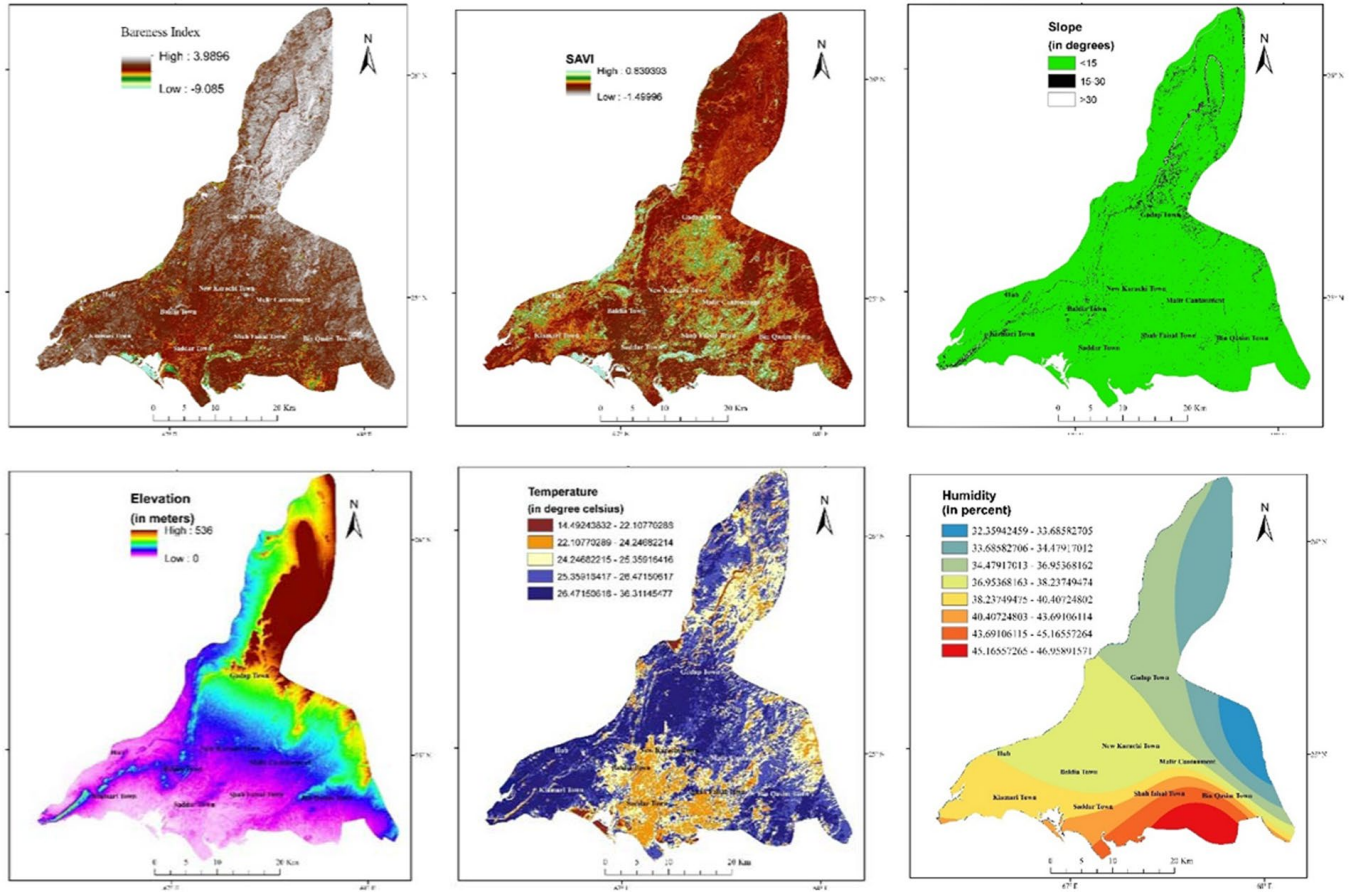
The spatial maps of variables used in site suitability model. (**a**) Bare land map of the study area, generated from Landsat 8 imagery (2018). (**b**) SAVI map of Karachi, generated from Landsat 8 imagery, 2018. (**c**) Slope map of study area divided into 3 classes according to suitability criteria of this sudy. (**d**) Elevation map of study area derived from ASTER DEM. (**e**) LST distribution map showing temperature in degree Celsius. (**f**) Map showing mean annual humidity of study area. *[Landsat-8 and ASTER GDEM images are courtesy of the U.S. Geological Survey]*.

The bareness index revealed some pockets shown in light colors (Fig. 3a) considered as possible soil for plantation. This index classifies the study area into rocky terrain (majorly in the northeastern and northwestern parts) and southwestern side of urbanized study area (shown with middle values, dark colors) which are not suitable soil for the plantation. Jatropha Curcas favors well-drained aerated sandy, gravelly and loamy soils^22^. It is unable to grow on some varieties of clay due to their poor permeability which preclude water content to reach the Jatropha Curcas roots. Hard or rocky land is usually given the least priority for Jatropha Curcas plantation. SAVI filter is applied which suggested the existing plantation in the study region. The existing soil adjacent to plantation map (Fig. 3b) depicts the pockets in peripheral zones.

The slope map of the study area (Fig. 3c) is classifying the region into three classes. The areas with slope less than 15° (green color) is considered highly suitable for plantation. The areas shown in black has a slope of 15° to 30° and is moderately suitable. However, any areas shown in white are considered least suitable with slope amount more than 30°. Surface slopes greater than 30 degrees directly influence the soil erosion, sediments transportation, irrigation and drainage patterns^23^. A land with slope greater than 25 or 30 degrees is usually given the least priority as it is a cause of water loss and soil erosion. The topographic elevation map of study area (Fig. 3d) presents the topographic features in Karachi. Overall, the surface elevation ranges from 0 (near to the coast, shown with light purple color) to 536 meters (northeastern side, shown with dark brown color). Jatropha Curcas is considered most suitable at an elevation of less than 1500 meters, considering this the whole Karachi area is highly suitable.

The land surface temperature is an essential factor of site suitability criteria. Jatropha Curcas does not survive in extreme low temperatures as it is sensitive to low or freezing temperatures^24^. An annual mean temperature below 15 °C can considerably lessen the yield of Jatropha Curcas It should be planted in areas with 17 °C to 28 °C mean annual temperatures. However, higher temperatures are not harmful for their growth, as Jatropha Curcasbeing a therophyte plant, can tolerate high temperatures^16^. The distribution of estimated LST (Fig. 3e) depicts a favorable range of temperature. The brown color symbolized the low LST over the water bodies, elevated land, near coast, whereas blue shades indicate relatively higher temperatures in the surroundings of urban sprawl (middle values of LST). The mean annual humidity of the study area is interpolated by Krigging method to determine the spatial trends of humidity. Three classes of high, moderate and low humidity are shown (Fig. 3f). The areas with high humidity are most suitable for Jatropha Curcas Most of the Karachi region shows a trend of relative high annual humidity due to coastal environment.

**Figure 4:**
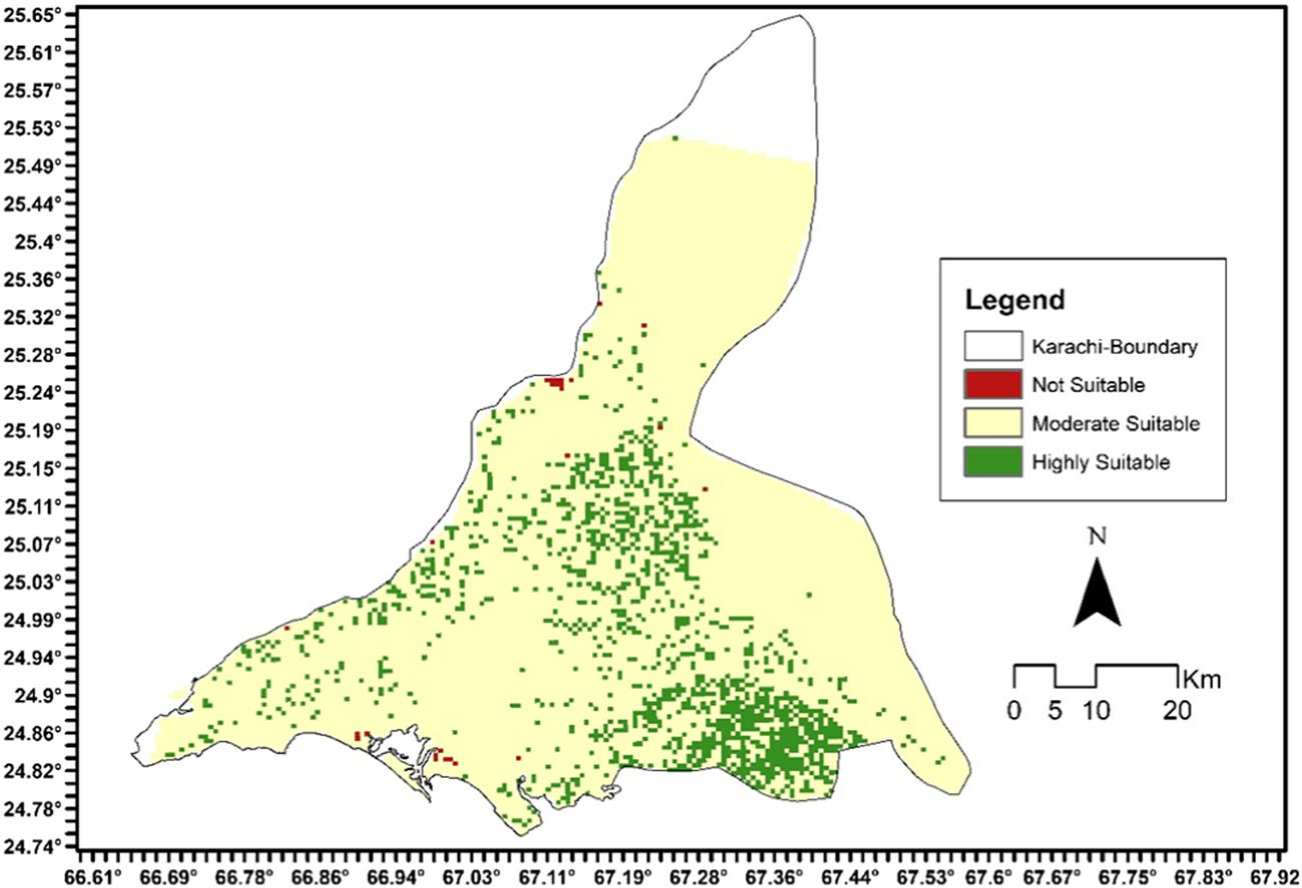
Suitability map for Jatropha C. plantation in Karachi.

An overlay map is generated after rigorous computation through a careful weighted overlay method Fig. 4 presents three classes of suitable land for Jatropha Curcasplantation in Karachi region. The weighted overlay analysis is an arithmetic function of variables including slope, humidity, SAVI, and LST. These layers were assigned a weightage of 25% each. The most suitable sites are shown in green, the beige color represents moderately suitable sites and the red color shows least suitable sites for plantation of Jatropha Curcas. The suitable sites are validated with bare land and precipitation network. Moreover, the resultant map is inspected by correlating the pilot project of Jatropha cultivation in eastern side (in a private farm house) of Karachi.

## Discussion

The plains of Pakistan host fertile soil and dominant in agriculture sector. Conventional energy resources are depleting in Pakistan. Pakistan has been blessed with a variety of natural energy sources (natural gas, crude oil, coal reserves, gas hydrates), renewable energy (biomass, hydro, wind, solar and tidal powers) but unfortunately implimentation on effective strategies yet need to be promulgated. Expending automobile industry, agriculture, heavy machinery industry, domestic, commercial and other sectors are major consumers of fuel energy in Pakistan. Pakistan imports different types of energy, mostly oil products to fulfill its energy demands byspending huge income. The growing demand of energy fuel from depleting conventional hydrocarbons can be balanced by apropos deputy energy sources such as biodiesel of Jatropha Curcas. It is also a medicinal plant known as “Jamalgota” and energy plant, which can yield biofuel from its seeds. Advancement in technological solutions inclined towards resources which are environmental friendly. We have inspected the Karachi land for plantation of Jatropha Curcas. It is high oil yielding plant, require fewer dependent inputs and payback high energy fuel from the degraded soil, where no fruit or high economic vegetable plants grow^25,26^. About 90 percent Jatropha Curcas plant is cultivated on the lands where annual precipitation is above 600 mm^27^. For a good production of the plant, the annual rainfall should be around 900–1200 mm otherwise an alternate irrigation source may be needed. However, the plant may grow in regions with annual rainfalls as low as 250 mm in case a high humidity is present in the air to supplement water deficit^5^. However, too much rain combined with high humidity may become detrimental for the plant, causing root or stem fungus. Jatropha Curcas can cultivate on a lower altitude from 0 to 500 meters amsl (above mean sea level), can also grow on high altitude area of 1800 m amsl, although, there may be a risk of frost attack at higher altitudes which can stunt the plant’s growth^20^. On the northwest of Karachi, there are two small ranges and between these hills wide coastal plains interspersed with river beds and water channels are situated. There are two non-perennial rivers, draining into the Arabian Sea after crossing the thickly populated city areas: Hub river (through western mountains), Lyari river (central plains of Karachi city) and the Malir river (north-east to the centre of Karachi city). Together, these three rivers make the Karachi watershed. Since the watershed is relatively small and due to extensive urbanization and ill-planned development projects. The river channels and connected small streams may presumably suggest the pathways for watering the plantation due to lack of ample precipitation. For this reason, alternate water sources might be considered. Jatropha Curcas plantation for the purpose of biofuel extraction in the Karachi would not only prove to be beneficial for the local economic prosperity but for environmental sustainability. The plant growth of Jatropha Curcas requires low nutrients, deficit water supply and less management since it is a drought resistant plant which subsidizes nitrogen content^15^, and favourable for plantation in Karachi. The Jatropha Curcas is successfully farmed in eastern parts of Karachi with constrained water and compost supply particularly in conditions where topsoil is free from a fear of water logging and the soil has free draining sands.

There are several million hectors of land available in Sindh, Punjab and Balochistan provinces. The eco-friendly and economically efficient Jatropha Curcas cultivation may be suitable in such areas which may help to reduce the consumption of the fossil fuels and elevate the green-economy. It is suggested that further land shall be judged as potential sites for Jatropha Curcas plantation in other parts of Pakistan. This multidisciplinary study highlights significant potential of bioenergy plants in Pakistan and broaden the scope of biotechnology, geospatial technology, soil sciences and environmental studies which reduce the chaos in plantation of Jatropha crop and may contribute in a sustainable crop management system. Thus, contributing in economic growth, creating environmental balance, and adding supplement in national energy demands.

## Conclusion

The present study showcases integration of meteorological and satellite images processed by ArcGIS tools. All required parameters were found to be favorable for plantation of Jatropha Curcas in Karachi. The study area is classified into three suitability classes. Highly suitable sites for Jatropha Curcas plantation lie in eastern Karachi. A field evidence of Jatropha Curcas plantation in some farm houses of eastern Karachi provided confidence to the results derived in this study. It is recommended that the aid of geospatial technology integrated with biotechnology and soil sciences will strengthen the cultivation of Jatropha Curcas crop in Pakistan.
